# “I Would Consult a Doctor, But What the Rabbi Says Goes”: Ultra-Orthodox Jews’ Relationships with Rabbis and Doctors in Israel

**DOI:** 10.1007/s10943-024-02014-7

**Published:** 2024-02-29

**Authors:** Rivka Neriya-Ben Shahar, Fany Yuval, Aviad Tur-Sinai

**Affiliations:** 1 Department of Communications, Sapir Academic College, Sderot, Israel; 2 Department of Public Policy and Management, Chairwoman, Guilford Glazer Faculty of Business and Management, Ben-Gurion University of the Negev, Beer-Sheva, Israel; 3 School of Public Health, University of Haifa, Haifa, Israel; 4 Department of Health Systems Management, The Max Stern Yezreel Valley College, Yezreel Valley, Israel

**Keywords:** Ultra-Orthodox Jews, Club theory, Listening guide, Medical decisions

## Abstract

We examine relationships among ultra-Orthodox Israeli Jews, their doctors, and rabbis when medical decisions are made. Analyzing excerpts from sixteen focus groups with 128 ultra-Orthodox Jews, we determine how their belief system affects their decisions about whom to trust and follow when the doctor’s instructions contradict the rabbi’s advice. We argue that the strict behaviors described here with regard to relations among doctors, rabbis, and patients, function as social capital that raises the status of ultra-Orthodox Jews as members of an exclusive club that balances health decisions with the social demand to obey their religious leaders.

## Introduction

“We canceled the examination we were supposed to have today,” said an ultra-Orthodox friend. “Why? Didn’t the doctor say the fetus might have a problem?” I asked. “Our rabbi said everything will be fine, so we relaxed and canceled the examination.” This conversation exemplifies the unique perceptions and attitudes of ultra-Orthodox Jews with regard to the triadic relationship that plays out in medical consultations among themselves as patients, their doctors, and their communities’ religious leaders and authorities—their rabbis. To explore these relations, we combined a socioeconomic theory with a psychological one.

Berman (2000) uses club theory to explain what may be seen as irrational behavior on the part of Israeli ultra-Orthodox communities. The communities’ remarkable mutual-insurance network, he found, compensates them for members’ strict religious practices. The ultra-Orthodox sacrifice time and money, among other things, to signal their strong commitment to the community and contribute to their group’s social capital (Bourdieu, 1986; Putnam, 1995). From another perspective, Gilligan’s (2015) listening guide is a sensitive qualitative method for analyzing interviews. Using it to understand the economic aspects of the costs and benefits of belonging to the group, we argue that the strict behaviors described here in relations among doctors, rabbis, and patients function as social capital that raises the status of ultra-Orthodox Jews as members of an exclusive club. They balance member’ health decisions—perhaps the most vital and critical decisions in life—with the social imperative of obeying their religious leaders.

Based on sixteen gender-separated focus groups comprising 128 ultra-Orthodox men and women, we explored several questions: Who guides the medical decisions of ultra-Orthodox Jews—their rabbis or their doctors? Under what conditions? In what order? Who has the last word? Bearing in mind that the ultra-Orthodox community is not homogeneous, we also traced recent dynamic changes in the community’s perceptions of the complicated relationship between doctors and rabbis. Our findings should be useful for those seeking to provide culturally sensitive services to any community.

## Literature Review

### Culturally Sensitive Care Within the Ultra-Orthodox Community

Culturally sensitive care is based on adaptations of healthcare treatments to sociocultural, linguistic, and religious backgrounds (Bilu & Witztum, 1993; Holt & McClure, 2006; Witztum & Goodman, 1999; Ypinazar & Margolis, 2006). Specifically, the intersection of religion and health care suggests that the beliefs of the mainstream healthcare system differ significantly from some patients’ attitudes and perceptions. One of the central insights of these approaches is that careful and sensitive listening to various discourses reveals alternative cosmologies of illness, health, curing, and healing (Kasstan, 2019; Yehya & Dutta, 2010).

We chose several interesting studies from the many relevant case studies that explore the intersections of religion, religious authority, and health care in various religious contexts. Religious leaders as health advisers are unique neither to Israel nor to the ultra-Orthodox community. People often seek religious leaders’ advice and guidance at times of uncertainty. Covid-19 was an opportunity to observe religious leaders’ actions (in various ways) as health promoters. For example, Viskupič et al. (2022), comparing the effectiveness of religious, medical, and political leaders in terms of their public-health messages about Covid-19, found that people tended to obey religious leaders even though the others’ messages were identical. Based on this study, Meyer et al. (2022) recommended that the U.S. healthcare system and pharmacists collaborate with religious leaders to overcome the hesitancy about vaccinations within Christian communities.

The need for trust in the healthcare system is especially critical when people must make decisions about issues over which they may have misgivings, such as getting vaccinated. Patients from minority groups may find it difficult to trust the healthcare system. For example, studies have reported that Arab mothers in Israel distrust doctors and the Ministry of Health. They distrust the doctors because the healthcare system meters patients’ “face time” with doctors, leaving scant opportunity to establish a trusting relationship with them. In addition, Arabs argue that the Ministry of Health gives them poor-quality service and partial information (Shahbari et al., 2020).

The Israeli ultra-Orthodox Jewish community provides an appropriate case study for the intersection of religion and health care. This strictly religious Jewish community comprises three main sub-communities: Lithuanians, Hasidim, and Sephardim (Jews originally from Arabic, Farsi, and Turkic-speaking countries). Together, they formed 13% of Israel’s population (Malach & Cahaner, 2022). Their religious and social life is bounded by the strict interpretation of *halakha*, Jewish religious law, and unquestioning faith in rabbinic authority (Brown & Leon, 2017; Soloveitchik, 1994). This community actively safeguards its cultural continuity by separating itself from other Israeli communities (Caplan, 2007; Sivan, 1991).

Ultra-Orthodox men are an intellectual “society of scholars” with a commitment to learning Torah (Friedman, 1991, 1993). Ultra-Orthodox women care for their relatively large families (seven children on average) while meeting many other communal needs that give them a very significant presence in the public sphere. With an average of 14 years of schooling, they work, study, and support their families, many as primary breadwinners (Davidman, 1991; El-Or, 1994; Fader, 2013). In recent decades, a change in ultra-Orthodox employment and education patterns has to the rise of an ultra-Orthodox middle class (Malach & Cahaner, 2022; Zicherman & Cahaner, 2012).

Many studies about ultra-Orthodox Jews and health care highlight the importance of culturally sensitive medical care. This community’s close and strict matchmaking system makes its’ members particularly sensitive to mental-health issues and special-needs children (e.g., Band-Winterstein et al., 2019; Birenbaum-Carmeli, 2008; Bloch et al., 2018; Feinson & Meir, 2012; Glasser et al., 2016; Golos et al., 2011; Greenberg et al., 2012; Shaked & Bilu, 2006) and routine genetic tests (Zalcberg-Block, 2014). Even though we collected the data in 2019, before the Covid-19 pandemic could even be imagined, our data could have predicted some of the ultra-Orthodox behaviors in 2020. The pandemic led to numerous studies about this community’s approach to the virus and the high rate of Covid-19 spread in ultra-Orthodox cities (e.g., Kalagy et al., 2021; Zalcberg & Zalcberg-Block, 2021). The various attitudes toward Covid-19 vaccination echo the complex relationships that ultra-Orthodox Jews have had in the past with other vaccinations (Blas, 2019; Keshet & Popper-Giveon, 2021; Salama et al., 2021; Zamir & Israeli, 2017).

The Israeli healthcare system is public and assures equal access to healthcare providers. Nevertheless, the existence of a complementary system of private and supplemental services, widely taken up by affluent Israelis but out of reach to those of lower income, has left the ultra-Orthodox with less access than others to services that the public system does not provide. The gap in services also traces to community behaviors. For example, ultra-Orthodox women’s health care rarely includes much preventive medicine. As a result, these women tend to be overweight, have poor nutrition, engage in little physical exercise, and postpone medical care (Leiter et al., 2019). The emphasis on modesty as the community defines it (Stadler & Taragin-Zeller, 2017; Taragin-Zeller, 2019) leaves women with little knowledge, especially about “immodest organs” (Zalcberg, 2009). They tend to have many children, which takes a toll on their bodies (Teman & Ivry, 2021). In addition, they have specific needs that doctors unfamiliar with the community may regard as irrelevant. For example, one ultra-Orthodox woman asked to reschedule a gynecological test due to matters of *nidda*, the halakhic category that deals with marital relations before, during and after menstruation; the doctor responded by terming her concern “nonsense” (Baum et al., 2017).

The importance of focusing on the ultra-Orthodox community transcends the community’s specific healthcare issues. The unique insights derived from observing relations between the authority of religious leaders and that of medical professionals (Ivry, 2010, 2013) and viewing them through the eyes of club membership (Berman, 2000) may help healthcare providers deal with their patients in a more culturally sensitive manner. Our investigation into tensions between a highly traditional community and its dealings with modern health care may provide insights into how the sides negotiate these tensions.

### Relations Among Patients, Doctors, and Rabbis

Rabbis are the religious authorities of the ultra-Orthodox community (Brown & Leon, 2017; Friedman, 1991). Based on the doctrine of *da’at Torah,* “the idea that rabbis have the intellectual ability and the authority to weigh in on all sorts of decisions” (Raucher, 2020, p. 45), their authority has expanded in recent times from religious questions to issues in almost every realm of life (Shaked, 2009). They make decisions about issues such as medicines and surgeries. One of the common medical issues in which rabbis are highly involved is fertility. They make decisions on individuals’ reproductive practices (Irshai, 2014) and family planning (Taragin-Zeller, 2018, 2019). Rabbis are also involved in the other side of fertility—infertility issues and treatments (Ivry, 2013; Ivry et al., 2011). Many have connections with Israeli government elements and other modern institutions (Caplan & Stadler, 2009).

There are no statistical data about how many people in Israel ask rabbis about non-halakhic religious matters. Weingarten and Kitai (1995), however, claim that consulting rabbis is far more common within the ultra-Orthodox community than among other Israeli groups. Inside the ultra-Orthodox community, Ivry (2010) reports that Sephardim and Hasidim are more likely than Lithuanians to ask their rabbis about such issues. Nevertheless, consulting with rabbis about healthcare issues is not unique to the ultra-Orthodox; it recurs among other traditional and secular groups in Israel (Keshet & Liberman, 2014a, 2014b).

People ask rabbis about non-halakhic issues, specifically health care, for many reasons. Some explain that doing so allows them to consult with someone outside the family and find solutions tailor-made for them (Raucher, 2020; Shaked, 2009). Rabbis can provide practical help because they work with ‘*askonim*, mediators who have strong religious knowledge and numerous healthcare connections (Kasstan, 2019). Patients receive moral and mental support (Ivry, 2010, 2013) and a sense of comfort and security (Prins-Engelsman et al., 2018) by consulting with rabbis. Women explain that rabbis help them to cope with the uncertainties of pregnancy by making them feel that they are not alone (Teman et al., 2011, 2016). Weingarten and Kitai (1995) indicate that secular Jews who consult rabbis feel much the same. Many maintain that they value rabbis’ advice because it is given on the basis of human considerations, not just the results of mechanical medical tests.

Thus, the relationship between patients and rabbis is far more complicated than a rather simplistic distinction between Western freedom (and perhaps science) and religious authority (Agrama, 2010). Fader’s (2017) suggestion of “spheres of authority” also reflects this complexity. Similarly, Taragin-Zeller (2021) argues that these diverse relationships are far from binary extremes of obedience and opposition; rather, they can be plotted on a continuum of perceptions and practice.

This complexity increases when we add medical authorities to the relationship between patients and rabbis. Ostensibly, these triadic relationships have many advantages for all principals. Patients enjoy what Ivry and Teman (2019) called “moral outsourcing,” which enables them to lighten their moral responsibilities by dividing the labor between rabbinic and medical authorities. Rabbis gain control of their followers’ access to the secular world with its threats and influences (Kasstan, 2019; Raucher, 2020). Doctors can offer medical care to a hard-to-reach population and help their patients get the best treatments (Raucher, 2020). Concurrently, doctors’ collaboration with rabbis helps them steer their patients toward the best decision, even if it is a challenging one (Ivry & Teman, 2019).

Nevertheless, these triadic relationships create rivalries, confrontations, and tensions (Ivry, 2013). They lead to various options that create a continuum from conflicts and refusal to collaborate to passive collaboration and very active collaboration (Ivry, 2010). Such conflicts between doctors and rabbis and refusal to collaborate occur on both sides. While many doctors say that they must maintain good relationships with rabbis for their patients’ sake, other doctors criticize rabbinical intervention, some accusing it of interfering with the professional care they should provide (Raucher, 2020). Some rabbis simply do not trust the medical profession and give advice that contradicts the doctors’ suggestions. In cases of conflict, patients may be harmed. Many ultra-Orthodox Jews side with their rabbis even with regard to medical issues (Shaked, 2009; Simhi, 2013).

The order of consultation is a crucial part of this conflict. Since conventional medical professionals are the primary health authorities of every modern country, ultra-Orthodox patients first turn to doctors for diagnosis and treatment. Soon after, they consult with their rabbis. This order of consultation leaves final decision in the rabbis’ hands (Shaked, 2009). Patients have cited numerous examples of situations in which the rabbi’s opinion was better than that of the doctor, who, they said, kept making mistakes (Shaked, 2009). Many ultra-Orthodox interviewees, including those in our study, like to tell stories about doctors’ mistaken diagnoses and rabbis’ prevailing, along with cases in which (mostly) mothers’ prayers lead to miracles (Teman et al., 2011).

Even close collaboration, too, is very complicated. What is supposed to be culturally sensitive medicine may become stereotypical medicine, creating ethical dilemmas. Doctors may collaboratewith rabbis not just for their patients but also to obtain more recommendations and referrals from them because it is good for business. Some doctors, for example, suggest that their patients ask rabbis or even send patients to get permission from rabbis without asking the couple if they wish to do so (Ivry, 2010; Raucher, 2020).

Rabbis may also have their own interests. Raucher (2020) suggests that their relationships with doctors amplify their authority in the community. Ivry (2010) argues that collaborations between the religious community and medical authorities have created hybrids such as kosher medicine—religious intervention in biomedicine—and medicalized halakha, “a growing tendency to think of medical interventions as imperative for observing God’s commandments” (ibid., p. 663). Ivry (2015) also coined the term “complex authority structure” to describe the rabbis’ strategies for dressing prenatal diagnoses in religious approbation, finding in them not only adaptations but also a situation of “religion expanding its relevance and authoritative reach by appropriating biomedical knowledge and enlarging its authority... *proactive efforts* to disseminate knowledge about the myriad reproductive technological options” (281).

Therefore, ostensibly friendly and culturally sensitive collaboration between rabbis and doctors may act against patients’ interests. Raucher (2020) argues harshly that rabbis’ and doctors’ relationships are based not only on mutual benefit but also on mistrust. Rabbis’ suspicion of medical professionals leads to more rabbinical intervention and oversight. Doctors overcome this mistrust for professional reasons but also for financial ones. Therefore, rabbis and doctors make decisions about ultra-Orthodox women’s reproductive health care but overlook the women. As medical and religious authorities try to attain their (paternalistic) goals, they compete for authority while benefitting from cooperation. The result is that both rabbis and doctors silence women’s individual experiences and the impacts of both authorities’ dealings them, sometimes resulting in women’s forgoing medical care. The best example is clinics with signs marking them as “kosher.” Women who see the certification may feel reassured but it also means that rabbis are minding their medical decisions (Raucher, 2020). Why do ultra-Orthodox patients comply with this situation? We argue that the answer is rooted in club theory.

### Club Theory and the Ultra-Orthodox Community

From a theoretical point of view, this complicated discussion about medical decision-making that focuses on the relationship between rabbis and patients may be part of the moral and epistemological conflict between science and religion (Evans, 2011; Evans & Evans, 2008). During the Covid-19 crisis, for example, consultation with religious leaders resulted in less compliance with national health rules (Taragin-Zeller et al., 2020). Many outside the ultra-Orthodox Israeli community were puzzled by this outcome. We maintain, however, that club theory may explain it.

“A club is a voluntary group deriving mutual benefits from sharing one or more of the following: production costs, the members’ characteristics, or a good characterized by excludable benefits” (Sandler & Tschirhart, 1997, p. 335). Iannaccone (1992) uses club theory to demonstrate how rational members of religious communities can benefit from what the outside world sees as strange behaviors, restrictions, stigma, and self-sacrifice. Seeing religion as a “club good” explains how “religious activities provide utility in proportion to the scarce resources devoted to them” (Iannaccone, 1992, p. 272). Iannaccone establishes a connection between the sacrifices the religious community demands and the success of religious groups. His rationale is based on the understanding that ostensibly unproductive sacrifices signal a commitment to the group and its collective nature and simultaneously function as a system for screening out free riders. The unconventional norms whet participation among members, who must sacrifice secular commodities to obtain the group’s help and support. Furthermore, as Kelley (1972) argues, strictness, costliness, and seriousness outperform content in explaining the success of religious groups, adding (Kelley, 1978): “The most revealing data about religious behavior... [is found] in actions that cost something in money, time, effort, anguish, involvement or sacrifice” (p. 168).

Berman (2000) used these insights to explain the seemingly irrational behaviors of voluntary poverty and high fertility rates among ultra-Orthodox Israeli communities: Club members benefit from access to a remarkably generous mutual insurance network, based on religiously motivated charitable acts.... Access to mutual insurance is excludable, making it a club good. Religious prohibitions can be understood as an extreme tax on secular activity outside the club, which substitutes for charitable activity within the club.... The increased stringency of religious practice is an efficient communal response to rising real wages. (p. 908).

Berman also notes the presence of “heterogeneous agents who signal their commitment to the religious club by incurring costs or ‘sacrificing,’ allowing the club to exclude free-riders, choosing only the most committed to the ultra-Orthodox community” (p. 909). Time and money spent are irreversible acts; therefore, they are sacrifices. Prohibitions are “forbidden behaviors, such as dietary restrictions...; sacrifice, in contrast, involves irreversible acts such as the destruction of resources” (p. 921).

Another aspect of club theory is maintaining continual tension with the secular society through stringent demands related to dress, food, and technology (Iannaccone & Berman, 2006). Grzymala-Busse (2012) showed how religious club goods create community networks for support. The system lowers the price of high fertility rates while expediting the community’s growth and raising the cost of leaving it. The consequences are “not only expansion and retention of members but also their continued strict doctrinal commitment” (p. 273).

In terms of health, ultra-Orthodox club goods (Berman, 2000) also provide benefits to the community. Members know that they will receive the community’s emotional, technical, and financial support in any health crisis. They also know that the unique relationships between rabbis and religious institutions and the healthcare system will give them priority and access to special treatments—very expensive club goods that enhance their well-being.

While many studies about the ultra-Orthodox community and health care have dealt with private aspects of medical decisions (even though rabbis and doctors were involved), our study focuses on the social opportunity to increase one’s religious and social capital by obeying the rabbis even in life-and-death decisions. We will show how the meaningful place of leaders in every religious community, and the power and control they wield over their followers, affect the group as a whole, not just as individuals. Our discussion takes the socioeconomic club theory a step forward by using it to explain crucial medical decisions. Moreover, we suggest that combining club theory with Gilligan’s (2015) approach to careful listening can create analytical tools with which to explore the complex relationships among patients, religious leaders, and medical professionals within religious communities.

The intersection of culturally sensitive health care; the complexity of the triadic relationships among rabbis, patients, and doctors; and club theory led us to the following research questions: How do the ultra-Orthodox perceive contacting their rabbis for medical consultation? Specifically, in which situations, or when, do they do this? What questions do they ask the rabbis about health issues? Who leads the decision-making process—the patient, the rabbi, the doctor, or two or all of them? How do the ultra-Orthodox perceive the complicated relationship between doctors and rabbis? In the next section, we describe the methodological tools that we used to answer these questions and the challenges we faced.

## Methods

To understand the perceptions of the ultra-Orthodox community about healthcare providers and services, we used sixteen focus groups, each composed of eight participants, 128 people all told. In accordance with ultra-Orthodox values, the genders were separated: eight groups for women only and eight for men only. To ensure representation of the community’s major subcultures, Hasidic, Lithuanian, and Sephardi ultra-Orthodox Jews were included (about a third from each subculture) in every focus group. Their ages ranged from 20 to 65 and most were married and parents (see Table 4, appendix). Hasidic Jews are members of a “court” whose leader, called a rebbe, is the focus of their world, a figure whom they habitually consult in every aspect of their lives. Unlike Lithuanians, Hasidic men as well as women often work outside the home. In contrast, the focus of Lithuanians is the yeshiva; hence, they are also known as “yeshivish.” Their lives center on the study of religious texts. Lithuanian men tend to be full-time students, their wives supporting the household. Finally, Sephardim generally have a focus that resembles that of Lithuanians but is flavored by their Middle Eastern and North African origins.

We had three research assistants, one man and two women. They invited the participants and facilitated the focus groups in accordance with our guidelines. While all the research assistants were Lithuanian ultra-Orthodox, they were deeply familiar with the other subgroups of the ultra-Orthodox community. We chose them on the basis of their professionalism and education, mainly because they had experience in group leadership and, in the case of the women, as research assistants in other studies.

The focus groups met in the winter of 2019, one year before the outbreak of Covid-19. While the pandemic changed the relationships between the ultra-Orthodox community and healthcare providers, we assume that in terms of the focus of this article, these relationships have returned to their pre-pandemic norm. We conducted our study in four geographic centers of the community: the two cities in central Israel that had the country’s largest ultra-Orthodox populations, Jerusalem and Bnei Brak, and two towns in more peripheral parts of the country that had totally ultra-Orthodox populations: Betar Illit and Modi’in Illit. There were two men-only groups and two women-only groups in each location (each group facilitated by a team member of the corresponding gender). The sample comprised those who defined themselves as ultra-Orthodox adults. We used snowball sampling, an appropriate strategy in studying people who generally prefer not to participate in academic studies. The diversity of the geographic locations and the inclusion of sub-communities and streams, together with consultation with both sexes (even separately), enabled us to obtain an overall picture of the participants’ opinions and perceptions. This approach also created an environment in which the participants felt free to tell their stories openly.

We chose the focus-group method because our main goal was to understand the perceptions of the ultra-Orthodox community about healthcare providers and services. Our questions covered many topics such as accessibility, prices, and availability of doctors, as well as cultural perspectives. Finding the issue of relations between rabbis and doctors so important and interesting, we devoted this article to this topic.

The choice of focus groups as the primary methodology for this project derived from our commitment to methodology, which is largely premised on the belief in the significance of social context to understanding the ways in which people attempt to make sense of their environment (Ackerly et al., 2006; Weiner-Levy, 2009). However, while the focus group is an appropriate forum for helping participants discuss and share their ideas and thoughts (Krueger, 2014; Warr, 2005), it is not the best method for sharing sensitive feelings. Had it been our goal to explore sensitive relationships among doctors, rabbis, and patients, we might have chosen personal interviews. Nevertheless, the data obtained were rich and insightful. The focus-group technique was beneficial in that it presented the ultra-Orthodox discourse on a group basis (Kook et al., 2019). However, it had the disadvantage of social pressure and the sub-optimality of a focus group for sharing stories of failure that other members may criticize.

Usually, the hardest challenge in studies such as this is gaining entry into the community. One of the authors, however, was raised in a strictly nationalist ultra-Orthodox family and retains many ties with ultra-Orthodox relatives and friends. Nevertheless, the participants were concerned about issues such as identification, values, modesty, and time*.* We understood that they might be troubled about the possibility of being identified by their responses or being asked questions offensive to them, their values, and their lifestyle. Other concerns might be the research assistants’ appearance in immodest dress and/or the excessive time that their participation in the research might demand of them.

Assurances of complete anonymity assuaged the first concern, about being identified. All participants were pseudonymized in order to ensure their confidentiality. They were asked to sign their names in initials and they disclosed neither their full names nor any other information that might identify them. This is why we cannot add specific information such as subgroups, age, and the number of children. We were able to resolve the second concern about values with a great deal of help from the ultra-Orthodox research assistants. They helped our team formulate the subjects and questions for the focus groups with sensitivity to language and values. Since the research assistants were themselves ultra-Orthodox, the participants could trust them to comply with norms of modesty and behavior during the group meeting. To provide an acceptable environment, the groups were held in one of the participants’ homes. They were assured that no responses would be deemed correct or incorrect.

As to the fourth concern, that relating to time, the research assistants faced challenges in arranging the groups. Many ultra-Orthodox men do not keep an appointment book for meetings and tend to arrange meetings on the day they are held (Hakak, 2016). Most women work full-time and have seven children on average. They do use appointment books but have little spare time. Thus, we scheduled the group meetings during the period called *ben ha-zemanim*, meaning during the yeshiva semester break. We also compensated the participants for their time by paying them ILS 100 (~ USD 28).

All the focus groups were recorded and transcribed. We subjected the qualitative data to thematic analysis using ATLAS software. From the hundreds of transcribed pages, we extracted only the theme of perceptions about relationships between rabbis and doctors. We translated the quotations from Hebrew to English with a particular eye to the dialect of the ultra-Orthodox. Gilligan’s (2015) listening guide proved invaluable in this regard.

Our results deal with three questions: When do the participants consult their rabbis about medical issues and what kinds of questions do they ask? Who leads the decision making? And what are their perceptions about the complicated relationship between doctors and rabbis?

*Ethical considerations*: Ethical approval for the study protocol was obtained from the Ben-Gurion University of the Negev Ethics Committee (Ref. Nr 1377-2). Before being interviewed in the focus groups, all participants signed an informed consent form explaining the aim of the study, their right to refuse or discontinue participation, and the confidential nature of the data.

The study was supported by research grant no. 18328 from the Israel National Institute of Insurance.

## Results

### When and What Type of Medical Questions Do They Ask the Rabbi?

The participants said that they consulted a rabbi about essential and exceptional issues. They used the terms “critical,” “significant,” “worrying,” and “checking results.” They described situations involving severe diseases and surgeries. Others talked about complex or uncertain treatments: “We ask for the rabbi’s advice if there is a surgery that needs to be done abroad”; when it is “unusual... not standard.” Some said they consult a rabbi if they had doubts about undergoing the treatment: “To have the surgery or not to have the surgery? If they suggest a test that I know is not routine... we do not know it very well.”

Other participants said the opposite. Their method was to consult a rabbi as a matter of routine. They used the terms “simple” and “usual.” “First, we asked whether to vaccinate the baby. The rabbi said yes. Then, we asked again after every birth.” The high fertility rates of the ultra-Orthodox yield many questions in this area. One man said: “I know someone who asked his rabbi about a birth. The rabbi said: ‘You should go to Shaare Zedek’” (an Orthodox hospital). Another participant said that the doctor recommended accelerating the birth “so we asked the rabbi what to do.” However, some participants said, with a bit of humor, that they would not bother the rabbi with minor issues: “When you have a headache, you don’t need to ask”; others said: “We don’t ask whether to get a strep test or take an antibiotic.”

The answers to the question “Which questions do you ask the rabbi?” fell into two main groups—halakhic questions, usually asked about whether a medical procedure or test accords with Jewish law, and non-halakhic questions, as shown in Table 1. Indeed, many participants agreed with (P1)’s response: “If it’s only a clinical issue, I would ask the doctor. If the issue is clinical and halakhic, I would ask the doctor and the rabbi.”

**Table 1:** Coding frame—when and what type of medical questions do they ask the rabbi?

Code name	Sub-code
When do they ask	
Essential	Critical, significant, worrying, unusual
Exceptional	Severe diseases, surgeries
Routine	Simple, usual medical procedures
What type of questions	
Halakhic consultation (Jewish law)	Pregnancy and childbirth
	Rules and prohibitions
	Checking results
	*Nidda—*gynecological problems
	Vaccinations
	Health care waiver
	Solutions that prevent going to the doctor
	Family planning and multiple children
Non-Halakhic consultation	
Medical literacy	Selection of a specialist physician
	Recommendations on professional doctors
	Selecting a hospital or where to get a medical examination
	Obtaining medical explanations and information
Support	Extraction of rights and knowledge about health services
	Shortening waiting times for a specialist doctor
	Psychological, spiritual, rabbi’s blessing
	Community support: financial support: Private medical procedures, drug costs; accommodation on Saturday near hospitals to avoid driving

Some women spoke about the halakhic question of needing to travel to a distant hospital before the Sabbath, during which halakha prohibits travel for matters other than life-and-death. (P2) and (P3) told stories about their children who were injured close to the Sabbath. Even if the children’s lives were in danger, their parents would be allowed to take them to the hospital but not to return home. (P2)’s rabbi told her to drive her injured child to the secular but more professional hospital that was farther away than the local Orthodox hospital, even if it meant that she would have to spend Sabbath there. (P3)’s rabbi said the opposite: He told her to go to the local Orthodox hospital even though the care there was not as good. Indeed, her daughter received suboptimal care. (P3) said: “The rabbis told me that it is *forbidden* to desecrate the Sabbath and that I must not travel. Had I known, I would have traveled and spent the Sabbath at the hospital.”

(P4) related to halakhic questions about a medical examination:

There was a medical examination that I had to do, and I had to get medicine. The doctor said, until you do the examination, you will not get the medicine. She said so explicitly and even wrote it. She said that this was their protocol. I went to another doctor in the same department. He said: “Take medicine. The halakha *forbids* this medical examination. I asked a rabbi, and he said that this examination is *forbidden at this time*. She [the first doctor] said to me—you should go to another doctor.

Another focus group suggested that doctors who work with their community should learn more about them:

(P5): You should teach them […] what is *allowed* and what is *prohibited* according to the halakha.

(P6): It is impossible to teach every doctor in the world what is *allowed* and *prohibited*!

(P7): They would not accept that!

(P5): Every medical examination is controversial. [(P5) is knowledgeable; he serves as a rabbi’s assistant in a public facility that people can approach for halakhic consultation]. In every examination, I will tell you what is *allowed* and what is *prohibited.*

A major topic in the men’s focus groups was questions related to *nidda.* “There are various kinds of gynecological problems. Sometimes, we need a rabbi’s decision about whether to do it this way or not to do it this way”; “In this context... of gynecology, sometimes the rabbis say, ‘It’s OK, you [the husband] can step out calmly; he [the doctor who will examine your wife] is OK.’”.

Another example concerns whether it is safe for a pregnant woman to fast for 25 h, as she would need to do on Yom Kippur and the Ninth of Av:

The gynecologist interferes! He wants to decide if she is *allowed* to fast on Yom Kippur or *not allowed*. The rabbis said that she must fast. However, he [the doctor] decided that he gets to decide. He knows that it is the routine opinion [that a pregnant woman] should not fast.

The men were also disturbed by women’s interrelating with male doctors: “Usually, women prefer to have a female gynecologist.... It is *prohibited* to go [to a male doctor].” While most women indicated that they preferred a female gynecologist, the men spoke from the halakhic perspective. The following quote provides an interesting insight into these relationships:

There are women whom the doctor tries to be friendly with. In the secular community, this is fine. In our community, it puts the women under stress. In our community, a woman is *not allowed* to laugh with a doctor. They [the doctors] need to take a course, have some training, on how to deal with our community.

This halakhic discussion is essential to the ultra-Orthodox social discourse (Neriya-Ben Shahar, 2020). The halakhic categories of “forbidden” (*asur*) and “allowed” (*mutar*) permeate not just the yeshiva but also the daily discourse in every ultra-Orthodox home. Nevertheless, given that the three main Jewish commandments concern observing the Sabbath, the rules of nidda*,* and the dietary laws, these topics are especially prominent in the halakhic discourse. The halakhic questions mentioned above, however, focus on the Sabbath and nidda only through the lenses of gynecological examinations and modesty issues. The participants mentioned the dietary laws in regard to some medicines but rarely raised issues about kosher food. Perhaps the reason is that the study was conducted in Israel, where all food served in hospitals is kosher although ultra-Orthodox patients in many secular hospitals rely on volunteers who provide food that meets stricter ultra-Orthodox standards.

Some participants explained that they would ask a rabbi whenever they needed to make a decision even where no halakhic issue is involved: “No one asks a rabbi about clinical or professional issues. The questions are about making decisions.” Some rabbis amass their abilities from experience: “Our rabbi also understands [using the word *meivin*, implying one who is insightful] medicine; “When he hears stories from people, he gathers lots of knowledge.” To prove their point, they also quoted the famous adage: “Experience is the best teacher.”

Some participants referred to the psychological or spiritual aspects of their inquiries:

In our Hasidic group, I think it’s mixed. . . . This is spiritual, not halakhic. There is a halakhic issue, and there is a rabbi’s blessing, and there is something spiritual. We call it spiritual consulting. A person comes to the rabbi and says: Listen, the results of the examination are…. The rabbi says: Don’t worry! The birth will be fine. You shouldn’t worry; God will help. It … gives him peace of mind.

### Ultra-Orthodox Perceptions About the Relationship Between Doctors and Rabbis

Ivry (2010, 2013) explores the variety of options in the triadic relations among patients, doctors, and rabbis, from conflicts via refusal to collaborate to passive collaboration and very active collaboration. Our participants’ perceptions of these relationships ranged from appreciation and collaboration to full-bore tension. The participants’ positive stories came mainly from doctors’ and rabbis’ collaboration on infertility issues. They agreed that in many cases, doctors appreciated rabbis and strongly recommended that their patients consult them. As (P8) said:

Some years ago, I had to get some fertility treatments. The doctor wasn’t ultra-Orthodox. However, every step she said to me: “If you want, go ahead and ask your rabbi.” She gave us the space to decide if we wanted [to do the treatment]. She understood us. Even though she wanted to do everything quickly, she gave us our space. She understood that we wanted to talk to the rabbi and take the step appropriately after knowing what to ask.

(P9) also indicated that even if the doctors themselves are not ultra-Orthodox, they care about the connection with the rabbi:

In matters of infertility, the doctor is cautious. He says, you can do this and that. Ask your rabbi about everything. He was fine, even though he was not ultra-Orthodox, and he was religious. He gave me a very, very good feeling. He is OK, not religious like us, but. . . .

This appreciation was mutual. Rabbis also expressed their acceptance and appreciation of doctors, as (P10) said:

My wife had to accelerate her childbirth, and the doctor said she had to do it now. I called Rabbi [a famous name]. He said: Why are you asking [me] if the doctor said something? He understands that this is something that should be done, and that’s that.

Facilitator: He reprimanded you for asking?

(P10): [Yes]. A pregnant woman, in her ninth month, needs to do an accelerated childbirth now!

Nevertheless, many participants sketched scenes of less collaboration and acceptance and more tension between rabbis and doctors, attesting to doctors’ criticisms of rabbis’ interventions:

(P11): Once, I went to the doctor after a rabbinic consultation about something usually not accepted in medicine. The doctor disregarded the rabbi’s instruction. He said: What? [When and where] do rabbis get to make decisions about these issues?

(P12): I heard something like that. The doctor said that when he starts to pray, rabbis will start to give medicines.

The women told similar stories. (P13) said: “Once, I said to the doctor that I wanted to have the rabbi’s advice. She said: ‘What about the rabbi? What on earth does he know if you are allowed to fast? Or about some examinations?’” (P14) tried to empathize with the doctor’s feelings:

It makes them nervous; I understand. Whatever [happens, the patient says:] “I’ll ask the rabbi.” You meet with your gynecologist and to everything she said, I answered, I’ll ask the rabbi.” She just wants me to do everything quickly.

The participants interpreted these tensions as part of the medical system’s lack of understanding about the ultra-Orthodox community’s values and perceptions. (P5) gave some examples from his experience as a rabbi’s assistant in a public facility where people can present halakhic questions in person or on the phone:

If the doctors decide that a woman should have a clinical abortion, perhaps this is their role. But let the woman ask her rabbi. . . . It’s not against the law! Let her ask her rabbi and don’t laugh at her! It’s not only abortions but also amniocentesis and embryonic protein tests [which are unacceptable in the ultra-Orthodox community]. They say very disparagingly: “The rabbi? Is he a doctor?” Why do you disparage her? Why do they hurt her feelings? […] She trusts her rabbi more than [she does her] doctor. It’s her right, and this is a democracy. . . . Or sometimes, the rabbi wants to talk to the doctor. Why doesn’t the doctor agree to talk to him? Why does he talk with the rabbi so haughtily?

(P15) told another story about a doctor’s lack of understanding of ultra-Orthodox values:

My wife was 42 weeks pregnant plus a few days. It was before the Sabbath, and we didn’t want to wait [for her to give birth] in the hospital. I talked with a rabbi who is also a doctor. He says, “Don’t trust me . . . but according to the results [of the tests], you can go [home] and come back after the Sabbath.” The department head was disgusted and just said: “Sign here and here. It’s a dangerous; you’re taking a risk!” They forced me to sign that I take responsibility and don’t know what I’m doing. . . . Some sensitivity! I take advice from my rabbi, and nobody wants to take risks about his child or his wife. Nevertheless, this is our community!

The first insight here is obtained by paying attention to who criticizes whom. Listening to the voices of tension (Gilligan, 2015), one discerns patients’ criticism of doctors and patients’ stories about doctors’ criticism of rabbis. We tried to find even one story about rabbis’ criticism of doctors, as Ivry (2010) reported, but could not (see Table 2). Perhaps a focus group is not the appropriate space to voice such criticism publicly; maybe, too, we should have asked about it directly. Perhaps the participants had no stories to tell about their rabbis’ criticism of doctors because the rabbis did not express any. Another possible reason is the rabbis’ awareness that they hold the decision-making power and, therefore, do not criticize the doctors, who are weaker than them.

**Table 2:** Coding frame—Ultra-orthodox perceptions about the relationship between doctors and rabbis

Code name	Sub-code
Collaboration	
Doctors	Flexibility of doctors
	Doctors' understanding of Ultra-orthodox society
	The professionalism of doctors
	The doctor recommends consulting a rabbi
	Doctors are cautious
Rabbis	Rabbis understand the necessity, urgency
	Rabbis support the doctors’ decisions
	The rabbi asks for the information from the doctor: diagnosis, opinions, advice, report
Full-bore tension	
Doctors	Criticisms of rabbis’ interventions
	The doctor disregarded the rabbi’s instruction
	The medical system’s lack of understanding of the community’s values
	Doctors despise rabbis
	Doctors disparage a patient's desire to consult a rabbi
	Doctors display a condescending attitude towards rabbis

The second insight is the freedom with which the men talked about women’s bodies. The ultra-Orthodox have many taboos about the body, specifically in reference to sexuality and private organs. This community is known for its strict modesty rules (Stadler & Taragin-Zeller, 2017; Taragin-Zeller, 2014; Yafeh, 2007). However, the men in their focus groups talked about women’s bodies in specifically gynecological contexts, telling stories about pregnancy and birth and even discussing the sensitive issue of birth control.

Raucher (2020) wrote critically about situations in which two or three men—a rabbi, a doctor, and sometimes a husband—talk about a woman as if she were not there as they take the liberty of making decisions about her life, body, and health. These sensitive stories, we argue, have a particular function in regard to membership in the ultra-Orthodox club. Men talked about their wives’ bodies because of halakhic questions connected with their personal stories. They were projecting their experience from the private sphere onto the public sphere. Having many children and, therefore, experiencing many births creates opportunities for multiple examinations and procedures and, therefore, for posing questions to rabbis. The outraged feminist reader may envision a suffering woman waiting for the rabbi’s answer to the doctor’s question as mediated by the husband. Among the ultra-Orthodox, however, this process is greeted with appreciation.

Indeed, if the men talked about women’s bodies, women also talked about men’s bodies. Our data included stories by women who told the group about husbands who did not want to be treated by female doctors and nurses. These stories revolved around issues of modesty and mixed-gender staff. Given that the stories assigned no role to rabbis, we omit them from this article. Nevertheless, while men gave themselves almost total license to talk about women’s bodies and pregnancy, arguing that they were doing so from a halakhic or medical point of view, talking about their own reproductive body remained taboo. Only one man spoke about his own body; he had asked his rabbi about a forbidden examination, perhaps connected to his reproductive system. Thus, men may be willing to talk about women’s bodies but not about their own in their halakhic discourse.

### Rabbi or Doctor: Who Leads the Decision Making?

Some medical decisions pertain to the short term, such as whether a pregnant woman should fast on Yom Kippur. Other decisions, such as undergoing surgery or taking fertility treatments, have farther-reaching consequences. Decisions about birth control may branch to matters of mental health (Taragin-Zeller, 2018, 2019). Nevertheless, when a rabbi and a doctor disagree, one of them must be wrong. When we put this question to the focus groups, *all* agreed that the rabbi should have the last word and that they would listen to him (see Table 3). They were convinced that the rabbi was always right.

**Table 3:** Coding frame—Rabbi or doctor: who leads the decision making?

Code name	Sub-code
Disagreement passes the decision to the rabbis	Last word, final decision
	Listen to the rabbi, follow the rabbi
	The doctor is mistaken, the doctor is wrong

(P16) said simply: “If I came to ask him [the rabbi], it means that I wanted to ask him what his final decision is. Otherwise, I would not have come.”

(P17): Once I went to the rabbi, and the doctor was really . . . totally opposed. He was very much against my having gone the rabbi and not to the doctor, that the rabbi took responsibility for this issue.

Facilitator: So, what did you do?

(P17): I followed the rabbi’s recommendation.

Facilitator: Is everyone alive? Are all of them healthy?

(P17): The doctor couldn’t help with anything.

The same was said in the men’s groups. For example:

Facilitator: If the doctor’s recommendation contradicts the rabbi’s recommendation, what would you do?

(P18): We would clearly take the rebbe’s [the Hasidic rabbi’s] recommendation.

(P19): If there’s a disagreement, obviously someone’s wrong. So, we take it for granted that it’s the doctor’s mistake.

(P15): If the doctor says [to accept a treatment because the patient’s condition] is life-threatening and the rebbe says don’t, we won’t. There’s no question here.

**Fig. 1:**
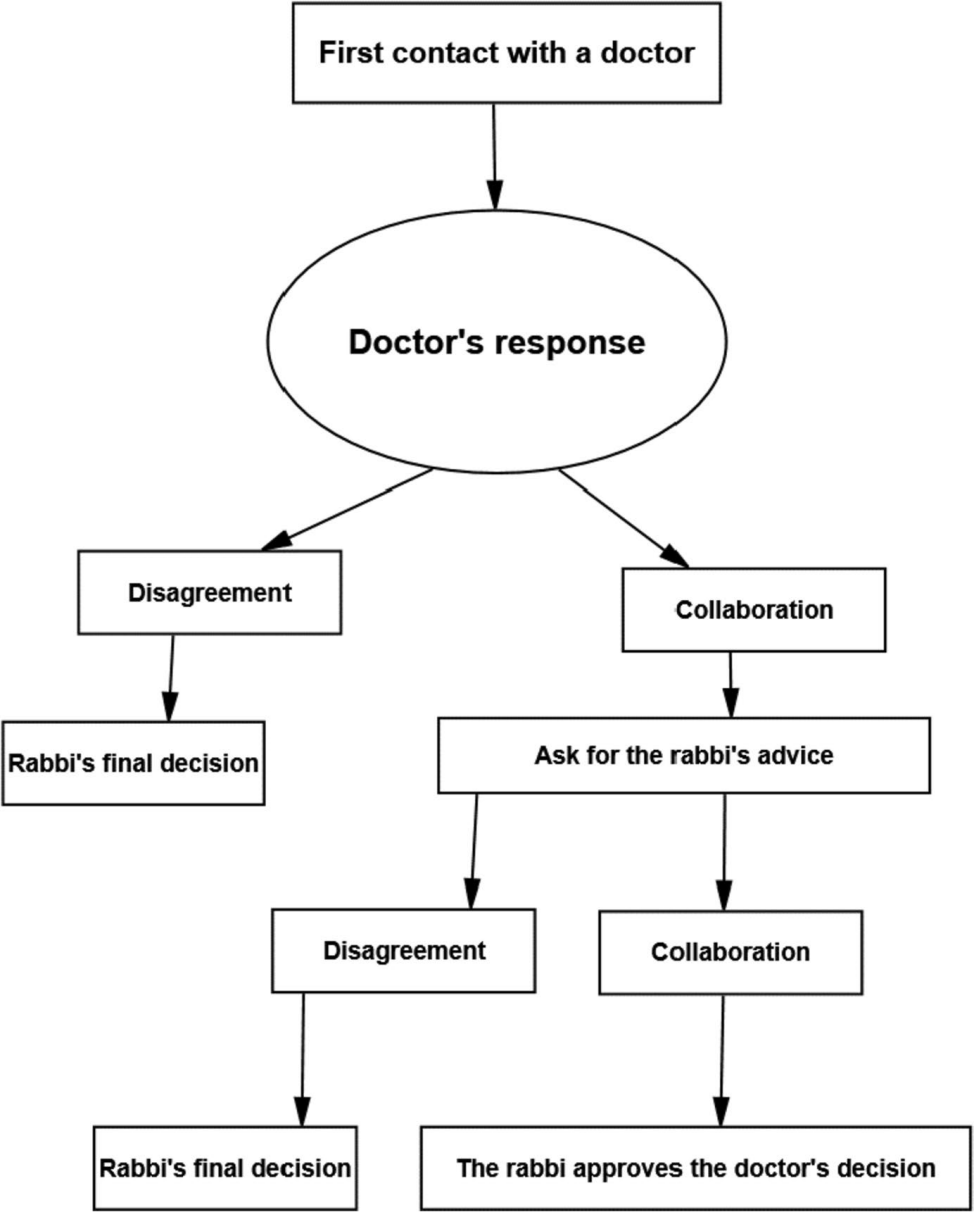
decision tree—Rabbi’s final decision

The order of contact also indicates whose word is final, as illustrated by the decision tree in Fig. 1 in a simple way.

Most of the participants said that the doctor is the first to be asked: “After the doctor’s recommendation, we go to the rabbi.” Some said that it depends on the urgency: “Depends on the issue. If it’s something that should be done immediately, we do it. If we can wait, we will ask the rabbi.” Some explained that they get the doctor’s report and then go to the rabbi. Women indicated that rabbis ask to see the doctors’ reports in order to give good advice:

(P20): I ask the doctor. The doctor gives me the report; with the report, I go to the rabbi.

(P21): The rabbi always asks what the last doctors’ opinions are.

(P22): He [the rabbi] looks at the examinations; if there is a need, he clarifies with medical ‘*askonim.*

The men described the collaboration:

(P23): You get the information from the doctor; then you go to the rabbi to ask him [what to do].

(P24): We do both—we take the doctor’s advice from the professional perspective; then we go to the rabbi.

Some interviewees said that they shuttle between the rabbis and the doctors. (P25) told the group:

Before surgeries, you should ask the rabbis. I asked about tonsils. The rabbi said to me: “Tonsils and polyps—I don’t know if [the surgery] is needed.” I asked another doctor, and he said it’s a borderline case. So I asked [another] rabbi….

The participants talked openly about their need to obtain both halakhic answers and moral and psychological support during the most stressful health difficulties. These aspects of the rabbi–patient relationship are not new (Ivry, 2010, 2013; Prins-Engelsman et al., 2018; Teman et al., 2011, 2016). Nevertheless, in the focus group, telling stories about these relationships had another important social function. Gilligan (2015) argues that listening to participants’ voices includes paying attention to several questions: “Who is speaking and to whom? In what body or physical space? Telling what stories about which relationships? In what societal and cultural frameworks?” (Gilligan, 2015, p. 69). At the next level, additional points are drawn out: “Who is there, who or what is missing, are there […] salient themes, striking metaphors or symbols, emotional hot-spots, gaps, or ruptures” (Gilligan, 2015, p. 71).

Addressing ourselves to questions of who is speaking and to whom, we argue that when people talk in a focus group, they appear to be talking to the facilitator and themselves. However, they are also talking to their communities. They take the opportunity to tell their personal stories in order to indicate that they are community members who accept the community’s rules. While ostensibly telling stories about their personal connections with rabbis and doctors, they are also talking about the ultra-Orthodox community’s relationships with the outside world. Moreover, via club theory we realize that they are also speaking of relations *inside* the ultra-Orthodox community, as to where and when people should ask for—and sometimes accept—rabbis’ decisions about life-and-death situations. Their methods of asking rabbis about medical matters, routine or unique, with or without connections to halakhic issues, are part of the price of club membership.

Thus, the fundamental questions of when, why, and in which order the ultra-Orthodox add rabbis to their relationships with their doctors represent basic social issues. Their responses to these questions show how they can increase their social capital by demonstrating their strictness. Their perceptions about prohibitions and forbidden and permitted examinations and procedures are not only part of their halakhic discourse but also symbols of their club membership.

Basing ourselves on Berman (2000), we argue that choosing to accept rabbis’ decisions is not just a personal decision but also a social interaction. This choice may entail a sacrifice even in cases that have a happy ending. Sacrifice “involves irreversible acts such as the destruction of resources” (Berman, 2000, p. 921). While the rabbi’s suggestion is tailor-made, personal, supportive, and helpful, it also takes much more time and effort and may be very confusing. Sometimes these decisions involve not just norms and bowing to social pressure but also real sacrifice. People receive support but pay for it by surrendering some of their own control and independence. This sacrifice symbolizes their commitment to the community. It also creates social capital and ensures cohesion within the community and between it and the world outside.

Our discussion also broadens the view from within the ultra-Orthodox community to the community’s relationships with doctors, the healthcare system, and the Israeli government. We maintain that there is a tension among that which is forbidden and prohibited, sacrifices, and reasonable costs and benefits (Berman, 2000). At least some doctors understand these multiple tensions and may attenuate or exacerbate them by their actions. The result may be a great deal of tension with rabbis and, at times, sacrifice on the part of patients. Sometimes the competition is not just between these sources of authority. The winner is the person who announces to the community that they were was heroic enough to sacrifice themselves or a family member just for having listened to the rabbi and reaffirming that it was God who saved them. All participants agreed that the rabbi has the last word, a stance that may lower the price of club membership. Sometimes, when the doctor supports the rabbi and encourages patients to consult him, the cost of the sacrifice may also decline, reducing its value. Then, ironically, the community must invest in an additional or a more significant sacrifice.

The public (governmental) Israeli healthcare system, combined with internal support among the ultra-Orthodox community, limits the price of the sacrifice. The government is also involved in this situation because if the rabbi supports a decision that harms the patient, the public healthcare system will pay the price. Berman (2000) notes that the government pays for non-working among ultra-Orthodox men and the numerous children of ultra-Orthodox women. Thus it maintains the tension between the ultra-Orthodox and mainstream society not only during crises such as the pandemic but also when very busy doctors, in the middle of expensive procedures in a crowded hospital, wait around for rabbis to give their answer. The risk to the patients is limited by the goods of the club and the knowledge that the community is always there to help them, be it by providing financial support for surgery abroad, sending meals and helping with childcare when the family is in the hospital, or offering spiritual and mental support.

### Independent Voices

Most of our participants indicated that whenever tension between doctors and rabbis arises, rabbis have the final word and people are willing to sacrifice themselves to be accepted into the club. Nevertheless, listening carefully, we heard some voices that expressed reservations [emphasis ours]: “**If** I go to a rabbi whom I can trust, so I trust him more than the doctor”; “**If** the rabbi **knows what he’s talking about**, I’ll accept his recommendations”; “I’d go to a rabbi **who knows** this subject”; “I’d **make sure** that the rabbi understands the question as clearly and correctly as possible.”

Some mentioned the option of seeking a second opinion from another rabbi: “I’d go to a more **highly regarded** rabbi.” “I’d ask the rabbi **again**, or I’d ask the doctor again.” “Some rabbis would say to you: go ahead, ask **one more rabbi**. This is my opinion but ask somebody else. Ask this rabbi; perhaps he’d say something else.” Another example, mentioned earlier, makes a similar point: “Depends on the issue. If this is something that should be done immediately, we do it. **If we can wait**, we will ask the rabbi.” (P3) talked about the rabbi’s order not to drive to a more professional secular hospital that was further away. “**Had I known,** I would have traveled and spent the Sabbath at the hospital.” We can hear some implicit criticism here. The rabbi should have known but did not. As a result, (P3)’s daughter will carry a scar (literally) for the rest of her life.

(P3)’s quote calls for more guided listening. Gilligan (2015) provides a more nuanced understanding of ambivalence. She argues that it arises not only in situations that yield discordant or harmonious “we” and “I” voices but also when there exists a “more complex understanding of... the sense of self within a shifting social/cultural framework.” The “had I known” voices reflect ambivalence. This ambivalence is inherent in the ultra-Orthodox community, which combines the use of modern tools, specifically modern medicine, with traditional behavior. The gap between Israel’s modern healthcare facilities and traditional life hides within these “If” voices. (P3) trusted the rabbi, chose the nearby traditional hospital, and carefully shared the results with the group. These “had I known” voices reveal the tension between medicine and religion, professional authorities and religious ones, obedience and independence, the community and the personal, and the public and the private. Medical and halakhic questions mingle everywhere.

The “had I known” voice becomes the “yes, I can” voice in Keshet and Popper-Giveon (2021), who write about ultra-Orthodox women’s antipathy to traditional vaccinations for babies (unrelated to Covid-19). They try to explain the women’s opposition to the vaccinations despite rabbis’ support of them. The women’s responses were varied: some interpreted the rabbis’ answer differently, others avoided consulting with rabbis. What stood out to us was this quote:

They claim that the instruction to vaccinate does not fall within the area of rabbinical expertise and cannot be relied upon because the rabbis do not conduct their own investigation and place their trust in medical knowledge. Unlike the rabbis, the interviewees conducted a thorough investigation into the matter, gathering information from online sources and social media networks, books, healthcare professionals, and other sources (p.1997).

As discussed above, the first claim is connected to areas that fall within the rabbis’ sphere of expertise—halakhic issues. The second claim intersects with one of the most crucial issues we have all experienced in the last several years—the debate over Covid-19 vaccination and the war between medical knowledge and “investigations” on social-media networks. These women in our study were not just hesitant or ambivalent. They displayed a great deal of independence, placing themselves, or at least their investigative abilities, above those of the leading authorities in their community, the rabbis.

These independent acts and voices are meaningful. They are small signs of criticism, deliberations, and independence. These few quiet voices actually mirror the situations of community members who sacrifice themselves and their voices. The small fissures in the prevailing self-assurance echo the complexity of naïve and straightforward heeding of rabbis.

Berman (2000) discusses “heterogeneous agents who signal their commitment to the religious club by incurring costs or ‘sacrificing,’ allowing the club to exclude free-riders, choosing only the most committed to the ultra-Orthodox community” (p. 909). This insight leads to the price of the sacrifice, which, we argue, is part of the price of membership that marks an ultra-Orthodox Jew as kosher. The members of the community pay the price whenever they ask a rabbi for a decision, even if they hedge their bets a bit by seeking out a rabbi whom they expect to give them the answer they want. When people choose the strictest option or ask the strictest rabbi, they symbolize their commitment to the community and increase their social capital.

The picture, however, is more complicated. Connecting Gilligan’s (2015) sensitive listening with Berman’s (2000) economic terms, we gained the insight that the relationships among the triangle of rabbis, doctors, and patients create a market—ostensibly a market in halakhic decisions. Freund et al. (2013) report that when ultra-Orthodox women had to decide on breast-cancer examinations, they consulted rabbis and regarded the latter’s decisions as indicative of God’s will. Nevertheless, they were “aware that different Rabbis can reach different decisions regarding the same medical procedure” (p.1083). Irshai (2014) reports that rabbis give different answers to different people for similar questions. Philip et al. (2010) and Coleman-Brueckheimer et al. (2009) present similar findings. They argue that the decision results from issues that arise in the consultation. How to frame the question and which rabbi to ask are crucial parts of the decision-making process. Taragin-Zeller (2021) calls this phenomenon “shopping around.” People move from one rabbi to another in search of the answer they prefer. Taragin-Zeller suggests that these daily healthcare decisions include not only acceptance but also negotiation and objection and the “shoppers” try to get the best rabbinic decision for themselves.

These economic terms undermine the simplicity of the rabbis’ decision. If everything the rabbi says is holy and right, why do believers shop around? The participants’ voice of ambivalence reflects their perceptions about what is prohibited and forbidden. Perhaps they regard the issues as nuanced rather than unequivocal.

## Limitations of the Study

Due to the characteristic features of the population investigated, our study suffers from several methodological limitations that kept us awake at night. Our decision to use focus groups as a primary methodology was guided by two main empirical challenges: gaining access to the relatively closed community under investigation and focusing on a sensitive topic that might trouble our participants as a potentially vulnerable collective. Aspiring to create a supportive atmosphere in order to motivate open discussion among the group participants and to obtain authentic rather than declarative answers, we had to facilitate a comfortable environment for our groups (Carey & Asbury, 2016). However, even though focus groups have been considered an important research tool for many years, their drawbacks and challenges have been extensively investigated (Barbour & Barbour, 2018). In this study, our main concern flowed from our commitment to preserving the social context within which our participants operate in their real lives, rooting the discussion in both dimensions—individual and group. However, since we were interested in the way men and women ascribe meaning to their experience as ultra-Orthodox patients, we were puzzled by the nuanced listening that revealed subtle ways in which these dimensions combined, since the collective construction of meanings and knowledge and the dynamic that emerges from the group may reflect the complexity of securing safe environments that may contradict the individual opinion—allowing us to draw a different understanding.

Sampling issues may also need to be addressed. Our sample was not statistical and was dictated by the need to establish gender-segregated groups. To cope with these limitations, we increased the number of groups and participants. This, however, abetted a profusion of meanings that coexisted among participants of one group and between the groups, sometimes connected to but on other occasions overlapping or even contradicting each other, expressing the multiple voices among the community but at the same time making it more difficult to come to a conclusion.

We were also unable to examine differences among ultra-Orthodox subgroups in reference to the research questions. As mentioned in the Methods section, our ultra-Orthodox research assistants recruited Hasidic, Lithuanian, and Sephardi participants. The participants’ anonymity was so essential for us that we did not know their names. The recordings gave us an indication of their gender and some participants mentioned their subgroup as they spoke. If the facilitator were to ask them to mention their subgroup (or their age or any other details about themselves), it would have badly interrupted the group’s streaming.

Payment issues may also need to be addressed, especially in research on low-income populations such that of our subjects. All these limitations show that there is room for follow-up studies on this topic.

## Conclusions: The Rich Use Their Gold Card to Shop Around; The Poor Sacrifice More to Remain in the Club

Combining the socioeconomic terms of Berman’s club theory (2000) and Gilligan’s (2015) guide to listening, we obtained a method for identifying the social, economic, and psychological factors that figure in the participants’ medical decisions. One of the insights revealed by this method is the differential nature of club membership. Indeed, sacrifice symbolizes the commitment to the community and creates and maintains social capital and cohesion inside and outside the community. However, following Irshai (2014) and Taragin-Zeller (2021), we may view the act of asking rabbis about health issues as a market. This market, like any other, has mechanisms that distinguish between rich and poor and between the advantaged and the disadvantaged.

We maintain that those who seek gold-card membership in the club can achieve it by embracing the sacrifice of accepting the strictest opinions. Those who have abundant capital can shop around for a decision that will provide them with an acceptable choice and leave their gold membership intact. Those with less capital may have to sacrifice more for the same level of membership. The rise of an ultra-Orthodox middle class in recent decades (Malach & Cahaner, 2022; Zicherman & Cahaner, 2012) may have exacerbated inequality within the community. The voices of ambivalence, minor signs of criticism, and independent deliberations may all be evidence of these changes.

Our method, we believe, will be useful for teasing out the issues that complicate the successful delivery of culturally sensitive healthcare in other insular communities. We maintain that the tools we developed in this study may be used to sketch the factors that affect how religious people elsewhere approach medical issues. Such a picture is vital for the delivery of culturally sensitive healthcare to insular religious communities in a manner that they are willing to accept and in a way that helps them.

## Appendix

See Table 4.

**Table 4:** Participant demographic characteristic, ultra-Orthodox Jews in sixteen focus groups (128 participants)

	Male (n = 64)	Female (n = 64)
Age, mean (s.d.)	30.4 (4.2)	36.3 (7.4)
Education status (percent)		
No education	4.0	10.0
Elementary education	8.0	6.0
High school education	66.0	54.0
Bachelor degree/diploma	22.0	30.0
Employment, percent	81.3	87.5
